# Prevalence of hepatitis E virus infection in wild boars from Spain: a possible seasonal pattern?

**DOI:** 10.1186/s12917-018-1377-4

**Published:** 2018-02-27

**Authors:** Antonio Rivero-Juarez, María A. Risalde, Mario Frias, Ignacio García-Bocanegra, Pedro Lopez-Lopez, David Cano-Terriza, Angela Camacho, Saul Jimenez-Ruiz, Jose C. Gomez-Villamandos, Antonio Rivero

**Affiliations:** 1 Infectious Diseases Unit. Instituto Maimonides de Investigación Biomédica de Córdoba (IMIBIC), Hospital Universitario Reina Sofía de Córdoba. Universidad de Córdoba, 2° Floor. Box 134.Avenida Menendez Pidal s/n, 14004 Córdoba, Spain; 2 Animal Health Department. Veterinary Science College, Universidad de Córdoba, 14014 Cordoba, Spain; 3 Animal Pathology Department. Veterinary Science College, Universidad de Córdoba, Cordoba, Spain; 4 Unidad de Enfermedades Infecciosas. Hospital Provincial, Complejo Hospitalario reina Sofía de Córdoba, Avenida Menendez Pidal s/n, 14006 Cordoba, Spain

**Keywords:** Hepatitis E, Wild boar, Prevalence, Seasonality, Foodborne

## Abstract

**Background:**

It has been shown that wildlife can serve as natural reservoirs of hepatitis E virus (HEV). The wild boar (*Sus scrofa*) is probably the main natural reservoir of HEV and could therefore represent an important route of transmission in Europe, especially in regions where game meat is widely consumed. We evaluated the prevalence of HEV infection in wild boar in the south of Spain, with the aim of identifying associated risk factors. A cross-sectional study that included hunted wild boar was carried out during the 2015/2016 hunting season (October 15 to February 15) in Andalusia (southern Spain). The outcome variable was HEV infection, defined as amplification of HEV RNA in serum by RT-PCR.

**Results:**

A total of 142 animals, selected from 12 hunting areas, were included and formed the study population. Thirty-three wild boars (23.2%; 95% CI: 16.8%–30.7%) were positive for HEV infection. Prevalence peaked in October and November, then gradually declined until the end of December. After multivariate analysis, only hunting date was independently associated with HEV infection across sex and age.

**Conclusions:**

Our study found a relatively high prevalence of HEV infection in wild boar in the south of Spain, suggesting that prevalence may depend on the season when the animal is hunted. In consequence, the potential risk of zoonotic transmission could fluctuate.

## Background

Hepatitis E virus (HEV) is an emerging cause of viral hepatitis in developed countries [1, 2]. The main route of transmission is the consumption of raw or undercooked pork, and pigs have been identified as the main host of HEV [3]. It has been shown that other animals, wildlife in particular, can act as natural reservoirs of HEV [4]. Among wildlife species, the wild boar (*Sus scrofa*) is probably the main reservoir of HEV [5] and could therefore represent an important route of transmission in Europe, especially in regions where game meat is widely consumed. In this respect, we recently described a familial HEV outbreak in our area that was linked to the consumption of wild boar meat, with a secondary finding in our analysis being a high prevalence of HEV in wild boar [6]. It has been proven experimentally that HEV-infected wild boar can transmit the infection to other animals, such as pigs [4, 7]. This plays an important role in countries where extensive pig farming is widespread, because it facilitates contact between domestic pigs and sympatric species and increases the risk of inter-species transmission. For this reason, the evaluation of HEV infection in wild boar and the identification of risk factors affecting transmission is important in order to determine the zoonotic potential of this emerging viral infection and enable control measures to be established.

Risk factors associated with HEV infection have barely been studied in humans. HEV infection has been associated with older males and certain genetic factors [8–10], although the reasons remain unknown. At the same time, living in certain regions has also been associated with a higher prevalence of HEV [11, 12]. Here we evaluated the prevalence of HEV infection in wild boar in the south of Spain in order to identify associated risk factors.

## Methods

### Study design and population

A cross-sectional study that included hunted wild boar was carried out in Andalusia (southern Spain) (36°N–38°600 N, 1°750 W–7°250 W) during the 2015/2016 hunting season (October 15th to February 15th). Age was determined on the basis of tooth eruption and animals of less than 12 months old were classified as juveniles, those between 12 and 24 months as sub-adults, and those over 2 years old as adults. All animals were classified according to sex. The sample size was calculated on the assumption that 10% of the samples would be positive for HEV. Hence, assuming a confidence interval of 95%, the minimum sample size was estimated at 139 animals.

### Variable collection and definition

A whole blood sample was obtained from all hunted animals by puncture of the cavernous sinus of the dura mater [13]. Serum was obtained from whole blood. Epidemiological variables were collected and included age, sex, date of sample collection, and hunting area.

The outcome variable was HEV infection, defined as amplification of HEV RNA in serum by reverse transcription polymerase chain reaction (RT-PCR).

### RT-PCR for detection of HEV

Viral RNA was extracted from 200ul of serum using the commercial QIAamp MinElute Virus Spin Kit (QIAgen. Hilden, Germany) and an automated procedure (QIAcube. QIAgen, Hilden, Germany). Samples were frozen at − 80 °C until analysis. For diagnosis of HEV infection, RT-PCR was performed using the LightCycler 480 system (Roche. Basel, Switzerland) described elsewhere [14]. For the reaction, the QIAgen One step PCR Kit (QIAgen, Hilden, Germany) was used. The primers (15 μMol) employed were: sense primer HEV5260 (5’-GGTGGTTTCTGGGGTGAC-3′) and antisense primer HEV5330 (5′-AGGGGTTGGTTGGATGAA-3′). The probe employed (20 μMol) was HEV5283 (5’-FAM-TGATTCTCAGCCCTTCGC-TAMRA-3′). The thermal profile was 50 °C for 30 min and 95 °C for 15 min, followed by 45 cycles of 94 °C for 10 s, 55 °C for 20s and 72 °C for 60 s. An external (in-run) standard curve was applied to calculate HEV viral load using a WHO Standard HEV strain supplied by the Paul-Ehrlich-Institute (code 6329/10).

### Statistical analysis

HEV prevalence was estimated from the ratio of positive samples to the total number of samples analyzed, with exact binomial confidence intervals of 95%. Throughout the study, we calculated prevalence by age and sex every week in order to evaluate the possible increase or decrease in HEV prevalence over time. Categorical variables were expressed as numbers of cases (percentage). Frequencies were compared using the χ^2^ test or Fisher’s exact test, and significance was set at a two-tailed *p*-value of less than 0.05. Bivariate analysis was carried out to discover the variables related to HEV infection, and multivariate logistic regression analysis was also performed. Analyses were carried out using the SPSS statistical software package, version 18.0 (IBM Corporation, Somers, NY, USA), GraphPad Prism, version 6 (Mac OS X version; GraphPad Software; San Diego, California, USA) and Winpepi software, version 11.36 (Brixton Health).

## Results

### Population

**Fig. 1:**
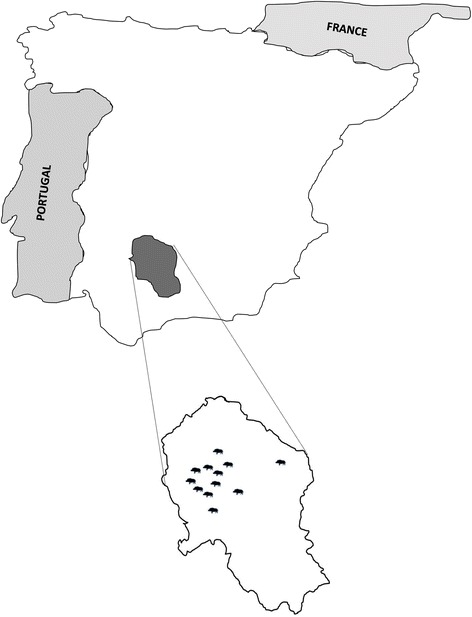
Hunting area sampling included in the study

A total of 142 animals were included and constituted the study population. These animals were selected from 12 hunting areas (Fig. 1). Sixty-four animals were male (45.1%) and 78 females (64.9%). Ninety-seven were adults (68.4%) and 45 non-adults (31.6%).

### HEV infection prevalence and associated factors

Thirty-three wild boars (23.2%; 95% CI: 16.8%–30.7%) were positive for HEV infection.

**Fig. 2:**
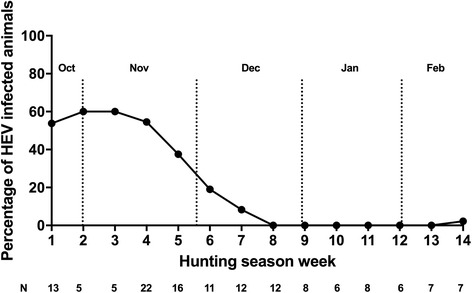
The prevalence of hepatitis E virus during each week of the hunting season (Oct 15 to Feb 15)

When prevalence was compared and analyzed according to sex, 20 males (31.2%; 95% CI: 21.2%–43.4%) and 13 females (16.7%; 95% CI: 9.9%–16.9%) (*p* = 0.047) presented HEV infection. No significant differences in prevalence were found between adults (25 of 97, 25.8%; 95% CI: 18.1%–35.3%) and non-adults (8 of 45, 17.8%; 95% CI: 9%–31.6%) (*p* = 0.394). An analysis of prevalence according to the week when the animals were hunted showed that it was higher in the first weeks of the study than at the end of the hunting season, February 15th (Fig. 2). Prevalence peaked in October and November, then gradually declined until the end of December. The prevalence of HEV infection varied between 60 and 0%, depending on the date of sample collection (Fig. 2).

By multivariate analysis, only hunting date was independently associated with HEV infection across sex and age (Table 1).

**Table 1:** Multivariate logistic regression model of HEV infection

Variable	Condition	*N*	HEV-infected	OR (95% CI)	*P*
Sex	Male	64	20	1.84 (0.69–4.92)	0.22
	Female	78	13	1	
Age	Adult	97	25	1.706 (0.55–5.23)	0.35
	Non-adult	45	8	1	
Hunting date	Oct 15 - Nov 15	23	13	44.73 (8.49–235.53)	< 0.001
	Nov 16 - Dec 15	49	16	28.09 (5.94–132.67)	< 0.001
	Dec 16 - Jan 15	78	3	1.03 (0.13–2.89)	0.99
	Jan 16 – Feb 15	34	1	1	

Legend: *N* number of animals, *HEV* hepatitis E virus, *OR* odds ratio, *95% CI* 95% confidence interval, *Oct* October, *Nov* November, *Dec* December, *Jan* January, *Feb* February

## Discussion

Our study found a 23.2% prevalence of active HEV infection in wild boars in the south of Spain. Interestingly, the prevalence varied significantly according to the period of the hunting season, with a higher HEV prevalence during the last weeks of October and the first weeks of November. This finding suggests a possible seasonal pattern for HEV infection in this species.

Studies performed in Europe have reported a variable prevalence of HEV infection in wild boar, fluctuating between 2 and 68%. A study performed in North Germany showed an HEV prevalence of 5.3% (10/189) [15], while in Central Germany it was 15.2% (7/46) [16] and rose to 68% in other areas [17]. Elsewhere, countries such as Italy and the Netherlands have reported a prevalence of 9.4% (6/64) and 8% (8/106), respectively [18, 19], while in Estonia and Hungary, the reported prevalence was 17.2% (81/471) and 12% (9/74) [20, 21]. Our results are consistent with those previously reported in Central Spain, where 27 out of 138 (19.6%) animals tested positive for HEV [22]. Our study shows an HEV prevalence of 23.2%. Differences between studies could be associated with various factors, including the sensitivity and specificity of the RT-PCR assay employed. These studies however did not consider external factors to explain the differences. Other studies evaluated risk factors associated with HEV infection in wild boar and identified host and environmental factors. Burri et al. studied 303 serum samples collected from wild boar killed between 2008 and 2012 in 10 different cantons in Switzerland [23]. That study reported a HEV seroprevalence of 12.5% and found that age (adults = 22.5%) and region of origin were factors associated with higher HEV seroprevalence [23]. Likewise, a study carried out in France found that the seroprevalence of HEV IgG antibodies was higher in the south (22.6%) than in the central part (9%) or the north (7.3%) [24]. In another study performed in Corsica, Jori et al. found that hunting season and age were risk factors for HEV seroprevalence [25]. Interestingly, hybrid wild boar showed higher HEV seroprevalence than pure wild boar and domestic farm pigs, suggesting they play an important role in the HEV reservoir [25]. Finally, using RT-PCR, Shielke et al. reported a higher prevalence of HEV infection in wild boars hunted in rural habitats than in urban areas [26], indicating that there may be a more efficient virus spread in wild boar populations in rural settings. Our study did not find that either age or sex were risk factors for HEV infection, a finding previously reported by others [27]. Our findings did however suggest that other factors, such as the season, could affect the rate of HEV infection in wild boar. In this respect, our study found that the major peak of HEV infection in wild boar was October and November, and then decreased significantly during the rest of the hunting season until February.

Seasonal patterns of HEV infection have previously been described in humans from Asian countries and are clearly linked to environmental factors, such as monsoons and floods, which have a markedly seasonal behavior. In this respect, one study conducted in China showed that the cumulative number of cases of acute HEV is concentrated in the cold season [28]. In studies carried out in India, Pakistan and Nepal, peak HEV infection is linked to floods during the monsoon season [29]. In these countries, the cumulative number of cases can easily be explained as due to the principal route of HEV transmission, which is fecal-oral [8]. Likewise, the authors of a study conducted in China that included farm pigs also described a seasonal pattern, with a major peak of HEV infection being reported in March–April in Eastern China, and a secondary peak in September–October in Southwest China [30]. Nevertheless, in countries where the main route of transmission in humans is via consumption of contaminated food, the seasonal behavior of the disease has not been well established and remains controversial. A study performed in the Southwest of England found that the highest number of cases occurred in the spring and summer [31], although the reason was unknown. By contrast, in another study carried out in France involving cases collected over a 5-year period, no seasonal variation in the number of cases of HEV infection cases was found at any time [32]. Our study suggests that there is a seasonal component in the prevalence of HEV infection in wild boar, with most cases concentrated in late autumn and gradually decreasing in early winter. This finding is striking and represents the first evidence of an environmental influence on HEV infection in a European country. The explanation for this, bearing in mind current knowledge of the epidemiology and pathogenesis of HEV, is unknown. An important point is that the lower prevalence rate in our study coincided with the reproductive season, which is usually between late November and January, when there is extensive contact between animals and the risk of transmission would therefore be expected to be much higher. Our study in fact found the opposite; there was a very low rate of HEV infection in this period compared with the pre-reproductive season. This could be explained by a route of transmission in wild boar that is as yet unknown, as well as by direct contact between the animals, and this may occur in the first weeks of autumn. It should also be mentioned that at the beginning of this period, extensive Iberian pig farming in the southwest of the Iberian Peninsula occupies agricultural land associated with hunting areas, which leads to increased animal population densities, and spaces and resources being shared with other wild animals. This favors an interspecies transmission of pathogens that needs to be elucidated. These points require further investigation.

Several limitations should be noted in this work. Firstly, our study evaluated only the prevalence of HEV infection in a single hunting season and a single region, and we were therefore unable to establish whether the seasonal behavior observed in our study can be extrapolated to other areas or later hunting seasons. Finally, we did not include other environmental or behavioral factors that may have influenced HEV prevalence in these animals, which may explain the seasonality found in our study.

## Conclusions

Our study found a relatively high prevalence of HEV infection in wild boar in the south of Spain. This finding suggests that the transmission of HEV infection to humans via meat consumption or contact with infected boar found in the wild may be an important factor. Nevertheless, our study suggests that prevalence may depend on the season when the animal was hunted, and the potential risk of zoonotic transmission may therefore fluctuate.

## Abbreviations

95% CI: 95% confidence interval
Dec: December
Feb: February
HEV: Hepatitis E virus
Jan: January
N: Number of animals
Nov: November
Oct: October
OR: Odds ratio
